# Border Control: The Role of the Microbiome in Regulating Epithelial Barrier Function

**DOI:** 10.3390/cells13060477

**Published:** 2024-03-08

**Authors:** Fernanda Schreiber, Iulia Balas, Matthew J. Robinson, Ghaith Bakdash

**Affiliations:** Microbiotica Ltd., Cambridge CB10 1XL, UK; fschreiber@microbiotica.com (F.S.); ibalas@microbiotica.com (I.B.); mrobinson@microbiotica.com (M.J.R.)

**Keywords:** microbiome, gut epithelia, barrier function, infections, inflammatory bowel disease, auto-immune diseases, metabolic diseases

## Abstract

The gut mucosal epithelium is one of the largest organs in the body and plays a critical role in regulating the crosstalk between the resident microbiome and the host. To this effect, the tight control of what is permitted through this barrier is of high importance. There should be restricted passage of harmful microorganisms and antigens while at the same time allowing the absorption of nutrients and water. An increased gut permeability, or “leaky gut”, has been associated with a variety of diseases ranging from infections, metabolic diseases, and inflammatory and autoimmune diseases to neurological conditions. Several factors can affect gut permeability, including cytokines, dietary components, and the gut microbiome. Here, we discuss how the gut microbiome impacts the permeability of the gut epithelial barrier and how this can be harnessed for therapeutic purposes.

## 1. Introduction

**Figure 1:**
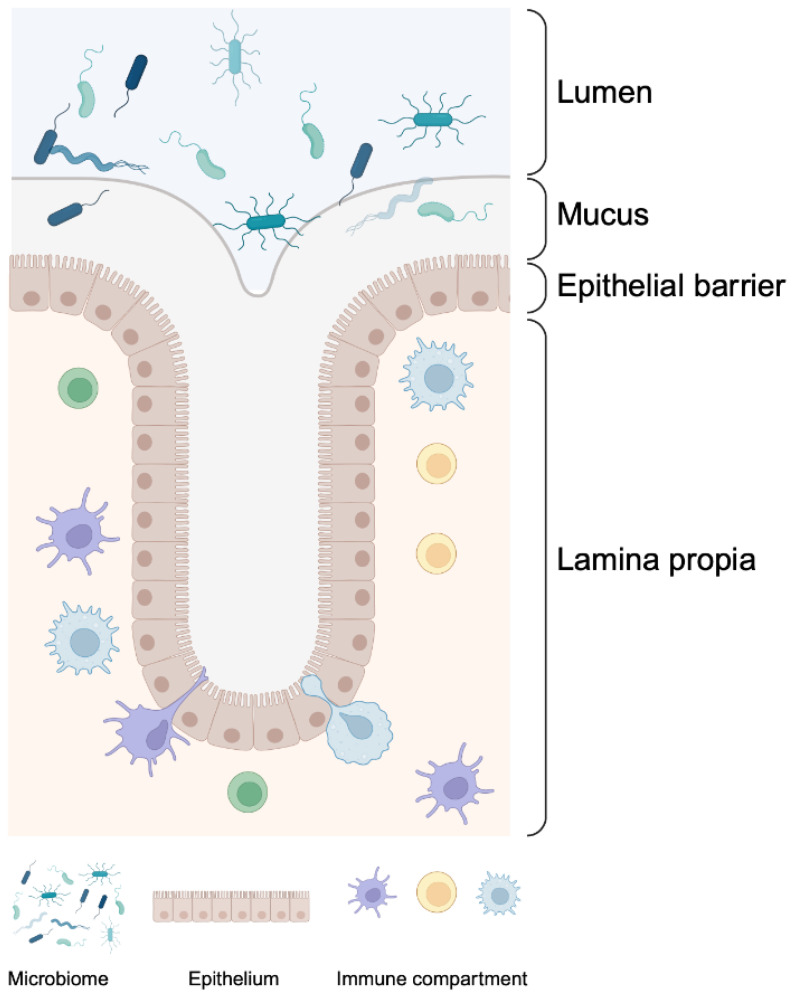
The intestinal barrier is a complex, multilayered system. The mucus layer acts as a filter, impairing access to the epithelial layer underneath it while providing nutrients to the microbiota residing within and around it. The epithelial barrier is made up of tightly packed epithelial cells forming a physical boundary between the interior and exterior of the body and regulates the passage of nutrients, water, and other molecules. Immune cells reside within the epithelial layer and under it, in the lamina propria, patrolling in close proximity to the epithelium and constantly monitoring for pathogenic invaders and testing environmental molecules. The interactions between these three components, the microbiome, the epithelium, and the immune compartment, regulate permeability across the intestinal barrier. This figure was created with BioRender.com.

The intestinal barrier is a complex system that provides a physical separation between the inside of the body and the external world. This barrier is not impermeable, but delicately balances the inward and outward passage of molecules. Despite the simplistic view of the intestinal barrier as a single layer of cells, it is made up of a complex multilayer system (Figure 1). The outermost mucus layer serves as a filter for potential pathogens in the gut lumen, while simultaneously providing a nutrient-rich environment for the microbiota residing within and around it. Underneath the mucus layer sits the epithelium, a cellular barrier defining the boundaries between the interior and exterior of the body. These tightly packed cells are responsible for the absorption of nutrients, the regulation of water transport, metabolite exchange and the exclusion of antigens and microorganisms [1]. This filtering capacity is not only due to the secretion of mucus but also to the regulation of bridges between adjacent microvilli, creating a mesh that keeps microorganisms away from the main body of the organ [2,3]. It also mediates the crosstalk between the local microbiome and the host’s immune system. Residing within and under the epithelium are immune cells that work in close proximity with the epithelium, scouting for pathogenic invaders and sampling environmental components from both diet and the microbiome. The interaction between these three compartments—the microbiome, epithelium, and immune system—regulates gut permeability [4]. Allowing the passage of nutrients, water and other factors needed for the correct functioning of the body, whilst excluding the translocation of harmful substances or even microorganisms, is critical for the wellbeing of the organism, and increased permeability or leakiness is often linked to diseases.

## 2. Key Elements in Barrier Permeability

**Figure 2:**
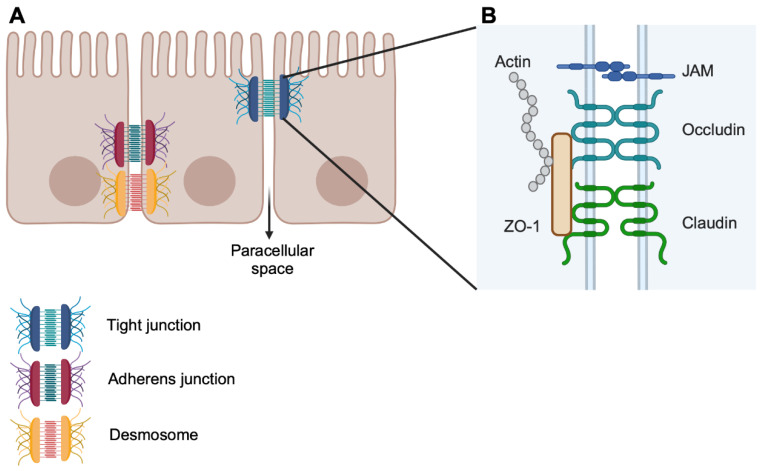
Epithelial paracellular transport. (**A**) Epithelial cells are held together through a series of protein complexes that breach the gap between cells and seal the paracellular space. Desmosome and adherens junctions are primarily involved in cell–cell interactions and bring adjacent cells together and promote the formation of tight junctions. Tight junctions seal the paracellular space and regulate the passage of molecules. (**B**) Composition of tight junctions. Claudins form the backbone of the tight junctions, and the composition of the claudins forming the junction determines the flow rate through the junction. Occludin and JAM proteins stabilise the junction. The intracellular component, ZO-1, provides a scaffold for the transcellular components and a link to the actin cytoskeleton. JAM: junctional adhesion molecules; ZO-1: zonula occludens-1. This figure was created with BioRender.com.

The intestinal barrier is primarily maintained by the epithelial layer, and considering the diversity of its functions, it is not surprising that there is no single mechanism that controls permeability. Indeed, there are multiple molecules and mechanisms involved in the transport and exclusion of different molecules and microorganisms. Transportation through the epithelium takes place through two major routes: the transepithelial or transcellular pathway, which involves the translocation of molecules through epithelial cells; and the paracellular pathway (Figure 2A), which involves transport through the space between epithelial cells [5].

Transport via the transcellular pathways is the physiological route for nutrient absorption and it involves specific transporters, such as for amino acids, sugars, short-chain fatty acids (SCFAs) [5], or it can occur in a non-specific manner through endocytosis (transcytosis) [6]. Although transcellular transport is important in the context of disease, in particular relating to diarrhoea, it has little impact on gut leakiness [7]. On the other hand, paracellular transport is regulated by intercellular complexes, which could belong to one of three types: desmosomes, adherens and tight junctions (Figure 2A) [5]. These complexes are located at the lateral membrane of the epithelial cells and keep the paracellular space closed.

Desmosomes are placed at the basolateral border and have intracellular and intercellular components. Intercellularly, desmosomal cadherins (Desmogleins and Desmocollins) provide the adhesive bond between cells. Intracellularly, the cadherins are anchored to desmosomal plaques, formed by the *Armadillo* proteins plakophilin and plakogoblin. This plaque is then linked to the cytoskeleton via desmoplakin [8]. Desmosomes provide resistance to mechanical stress [9], but they are also involved in managing intestinal permeability by creating strong cell–cell bonds and stabilising tight junctions [10,11].

Moving up apically along the lateral membrane are the adherens junctions. The primary role for adherens junctions is to mediate cell–cell adhesion, as well as taking part in cell signalling and transcriptional regulation [12]. Like desmosomes, adherens junctions include a cadherin family member as an intercell adhesion component anchored in the cytoplasm by *Armadillo* proteins. E-cadherin dimers bind homotypically to dimers on neighbouring cells. This is a weak cell–cell interaction that kick-starts the formation of the adherens junctions. Clusters of cadherins are then formed and spread, strengthening the bond between cells [13]. Intracellularly, E-cadherin binds to p120-catenin and b-catenin. These proteins provide a key link to the actin cytoskeleton and are therefore involved in cell motility and provide the plasticity needed in the regulation of cell–cell interactions [14]. Desmosomes and adherens junctions provide adhesive and mechanical features that stabilise cell–cell contact and lead to the assembly of the tight junctions [15]. Sitting close to the apical border, tight junctions seal the paracellular space and are the primary regulators of flux through the epithelium [13,16]

As with the other types of junctions, tight junctions have a transmembrane component and an associated cytoplasmic component (Figure 2B). The transmembrane component comprises two types of transmembrane proteins, namely claudins and occludin [14,17]. Claudins form the backbone of the tight junction by binding to each other in *cis*, forming rafts or strands. These strands then bind in *trans* to strands on the other side of the intercellular space, sealing the gap between cells [18], with occludin playing a stabilising role in bicellular junctions [14]. Junctional adhesion molecules (JAMs) also play a role in supporting the formation and maintenance of tight junctions and, together with occludin, stabilise the junction [19]. Sitting below the transmembrane proteins, zonula occludens proteins like ZO-1 and ZO-2 crosslink the tight junction, creating a scaffold connecting the transmembrane component to the underlying actin cytoskeleton [14].

Two tight junction-dependent pathways regulate transport across the gut epithelium: the pore and leak pathways. A third route, the unrestricted pathway, allows the passage of material due to breaching of the epithelium and involves epithelial cell damage or death [17]. Pore pathways allow the passage of molecules up to 0.6 nm, and this is dependent on claudins [17,20]. There are 27 members of the claudin family, and they can be divided into barrier and pore claudins, depending on their ability to increase or decrease transepithelial resistance, a method of assessing barrier function by measuring the flow of an electrical current through a cellular monolayer [21]. Pore-forming claudins increase permeability and include claudin 2, claudin 7, claudin 10 and claudin 15. Barrier-forming claudins, on the other hand, decrease permeability, and this group includes claudin 1, claudin 3 and claudin 5, among others [22,23]. In some cases, depending on the coupling between claudin, barrier forming claudins can act like pore claudins and increase permeability, with an example being claudin 4/claudin 8 coupling [22]. The localisation of claudins varies along the intestinal villi, with barrier claudins more abundantly found towards the tip of the villi and pore claudins found around the crypt area [24].

The regulation of permeability through the pore pathways is achieved through the regulation of claudins. Both pore and barrier claudins are regulated by the same factors, although usually in the opposite direction [25]. In fact, pore-forming claudins are upregulated, whereas barrier claudins are downregulated, in diseases where epithelial barrier integrity is compromised. In particular, claudin 2 upregulation has been associated with many pathologies linked to leakiness, as this pore-forming claudin also regulates water transport [17,26,27].

The leak pathway is not as well understood as the pore pathway. Although they do share molecular components, the mechanism behind them is different. Whereas the pore pathway transport mechanism is centred on the transmembrane component of the tight junction (claudins), permeability is regulated by the intracellular component in the leak pathway [17]. Flow through the leak pathway happens via the breaking and annealing of claudin strands, meaning the claudins on either side of the tight junction are pulled apart and there is a transient, localised break in the barrier [27]. Tension is regulated by the actin cytoskeleton through the branching of F-actin and the contraction of the perijunctional actomyosin ring [27]. ZO-1’s ability to bind both F-actin and claudin highlights its key role as a regulator of the leak pathway. Indeed, the absence of ZO proteins or modifications on the actin-binding site drastically alters barrier function [28]. However, the most well-studied effect of the actin cytoskeleton on permeability involves the regulation of the contraction of the perijunctional actomyosin ring by myosin light-chain kinase (MLKC) [27,29]. MLCK proteins phosphorylate the myosin light chain (MLC), causing a contraction of the ring, leading to a disruption in tight junction [17,29]. The transmembrane component does contribute to the leak pathway (claudin post-translational modifications and occludin expression can alter permeability by modifying strand strength and stability), but it is not the driving factor [27,30].

As mentioned previously, gut epithelial permeability is regulated via a three-way interaction between the epithelium, the microbiome, and the immune system. So far, we have introduced the molecular players involved in keeping the epithelial cellular barrier together and managing the passage of substances through it. We will now look into how the intestinal microbiome influences gut permeability.

## 3. Regulation of Barrier Permeability by the Gut Microbiome

**Figure 3:**
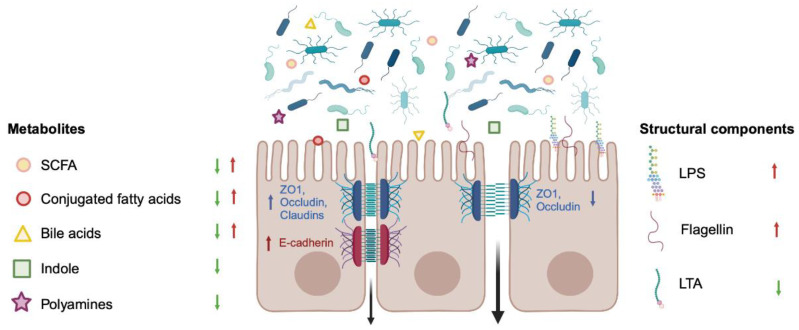
Regulation of epithelial barrier permeability by the microbiome. Bacterial metabolites secreted by the microbiome as well as bacterial structural components can regulate epithelial permeability. Structural components like LPS or flagellin have a negative effect on barrier function, increasing permeability (red arrows), whereas secreted metabolites like indole or polyamines reduce permeability (green arrows). Some metabolites, like SCFAs, can improve or worsen barrier function depending on compound or dose. These metabolites regulate permeability by modifying the expression and/or localisation of tight junction or adherens junction proteins. LPS: lipopolysaccharide; LTA: lipoteichoic acid; SCFAs: short-chain fatty acids; ZO-1: zonula occludens-1. This figure was created with BioRender.com.

The gut microbiome is an important component of the intestinal barrier, not only fending off pathogens by competing for resources and producing antimicrobial compounds but also by directly regulating hosts functions through the production of metabolites. Several microbiome-derived metabolites have been shown to impact barrier permeability (Figure 3) [31,32,33,34]. For instance, butyrate, a SCFA, can improve barrier function by facilitating the assembly of tight junction proteins and inducing the expression of claudin 1 in cell line models [31,35]. Other SCFAs, like propionate, can also induce the expression of tight junction proteins like ZO-1 and occludin and thus regulate paracellular transport [36,37,38]. However, at high concentrations, butyrate can cause a barrier breach, probably due apoptosis induction [31,39]. Moreover, conjugated fatty acids are produced by some intestinal bacteria (*Bifidobacterium*, *Butyrivibrio*, *Enterobacter*, *Roseburia*, among others) in the presence of a fat-rich diet. Conjugated fatty acids, like conjugated linoleic acid (CLA), can cause the redistribution of ZO-1, occludin and claudin 3, increasing paracellular permeability, as observed in a Caco-2 assay [40]. However, in in vivo models of colitis, CLA shows a protective effect and induces the expression of tight junction proteins, increasing barrier function [41,42].

Germ-free (GF) mice have been shown to display increased gut permeability compared to conventionally raised mice. This observation has been attributed to the significantly lower concentration of indole and indole derivatives in GF mice. This was associated with lower expression of both adherens and tight junction proteins (claudin 1, occludin, ZO-1, E-cadherin) [31,43]. It has also been shown that indol-3-propionic acid regulates the expression of occludin, ZO1 and claudin 1 in an in vitro cell line model as well as an in vivo rat model, resulting in reduced permeability [44,45].

The role of bile acids in regulating permeability is not clear, as there are reports of some bile acid metabolites having both a positive and negative effect on barrier function, depending on the type of bile acid, concentrations tested, and cell type used for the study [31,46]. Bile acids like chenodeoxycholic acid (CDCA) and ursodeoxycholic acid (UDCA) can regulate permeability via occludin phosphorylation, ZO-1 expression, and tight junction rearrangement, but whereas CDCA has a negative effect on permeability [46,47], UDCA promotes the expression of occludin and claudin 4, increasing barrier function [48].

Polyamines are metabolised from the diet by the gut microbiota, but also produced by the host in the small intestine [49]. Polyamines increase barrier function, controlling permeability not only through the regulation of tight junction proteins like occludin and ZO-1 [50,51] but also by promoting the expression of E-cadherin [52,53]. It is not only the secreted bacterial products that can have an effect on epithelial barrier permeability; rather structural components like lipopolysaccharide (LPS), flagellin or lipoteichoic acid (LTA) can also regulate barrier function via Toll-like receptor (TLR) activation, although with different outcomes [31].

The activation of TLR2 with ligands like LTA has been shown to reduce gut permeability and induce the expression of tight junction proteins in vitro [54] and preserve tight junction assembly both in vitro and ex vivo in a mouse dextran sodium sulphate (DSS)-dependent colitis model [55]. On the other hand, activation of TLR4 and TLR5 by LPS and flagellin, respectively, results in increased permeability and altered expression of tight junction proteins, both in vivo and in vitro [56,57,58].

Breaches in barrier function and increased permeability can lead to translocation of bacteria or bacterial products, which can in turn activate the immune cells patrolling the lamina propria. Many diseases have been linked to altered permeability and we will now explore how permeability can be both a result and a trigger for the pathological state and how the microbiome can contribute to ameliorating it.

## 4. Infections and Gastrointestinal Permeability

Increased intestinal permeability is a hallmark of gastrointestinal (GI) infections. This is due to many enteric pathogens actively disrupting the intestinal epithelial barrier [4,59,60,61,62,63,64] by altering the structure and function of gut epithelial tight junctions [4,59,65] (Table 1). Various infectious bacteria, viruses, parasites, and fungi dysregulate tight junctions by means of different mechanisms, including the degradation of specific tight junction proteins, the activation of host cell signalling pathways and the alteration of cell cytoskeleton [60,66,67].

### 4.1. Tight Junction Protein Degradation and Reorganisation

The direct targeting of tight junction proteins can lead to impaired junctional barrier structure and function caused by virulence factors either being secreted in the GI tract or situated on the external layer of the pathogen. These enterotoxins can degrade the tight junction by exerting a proteolytic effect, for example *Campylobacter jejuni* serine protease high-temperature-requirement protein A (HtrA), specifically cleaving occludin and E-cadherin proteins, thus breaking the cell-to-cell junction and leaving the basal epithelial layer vulnerable to bacterial invasion [68,69]. Other enterotoxins can interact directly with tight junction proteins as reported for *Clostridium perfringens* enterotoxin binding to claudin 4 or *Entamoeba histolytica* with claudins 1 and 2, leading to tight junction destruction [68,69,70,71,72,73,74,75].

Tight junction barrier structure and function can be altered quite dramatically by *Vibrio cholerae*, the well-known etiologic agent of cholera, a life-threatening infection characterised by acute diarrhoea. *V. cholerae*-derived cholera toxin subunit A (CTA) is responsible for adenylate cyclase activation and the subsequent increase in cyclic adenosine monophosphate (AMP) levels and opening of chloride ion channels, resulting in the substantial leakage of electrolytes and fluids (diarrhoea), characteristic of *V. cholerae* infections [76]. Strains lacking CTA are still able to induce diarrhoea, but with a less dramatic presentation. It is known that *V. cholerae* produces another tight junction-altering toxin that affects ZO-1 morphology, called zonula occludens toxin (ZOT), which targets barrier permeability specifically in the small intestine [77,78]. Another *V. cholerae* toxin targeting cell-to-cell junctions is hemagglutinin protease (Ha/P), a metalloproteinase with two functions, namely being substrate-lytic and a CT subunit A activator. This protease causes leakiness by degrading the transmembrane protein occludin, leading to the rearrangement of ZO-1 and F-actin morphology [59,78].

### 4.2. Activation of Host Cell Signalling Pathways

Microbial pathogens can also increase intestinal epithelial permeability through the modulation of signalling pathways associated with the tight junction complex. Changes in the gene expression of tight junction proteins and post-transcriptional events of key pathways like myosin light-chain kinase (MLCK), RAS homolog (Rho) and protein kinase A (PKA) can increase GI permeability. For example, the enteropathogenic *Escherichia coli* heat-stable enterotoxin A binds to extracellular domains like guanylate cyclase receptor (GC-C), triggering cyclic guanosine monophosphate (cGMP) formation and cAMP-dependent-protein kinase A (PKA), contributing to occludin’s rearrangement into the cytosol [79]. *Shigella flexneri* was also demonstrated to modulate the extracellular signal-regulated kinase 1/2 (ERK1/2) pathway, triggering alterations to the phosphorylation of claudins 2 and 4 as well as the occludin and ZO-1 proteins, resulting in altered barrier function and increased interleukin (IL)-6 and IL-8 [78,80]. Both *Camplylobacter concinus* zonula occludens toxin (ZOT) and *E. coli* heat-stable toxin B display similarities with *V. cholerae* ZOT and probably share regulatory pathways of the TJ complex [79,80,81].

Viruses, such as *Adenovirus*, *Rotavirus* and *Coxsackievirus*, target cellular receptors of the tight junction complex [82]. Both *Coxsackievirus* and *Adenovirus* target, as the name suggests, the coxsackievirus and adenovirus receptor (CAR), a transmembrane protein that, upon binding to the virus, triggers a molecular cascade leading to occludin internalisation within macropinosomes with subsequent tight junction disruption [81,82]. Although *Coxsackievirus* does not induce major tight junction disruption, additional viral interactions with the epithelial protein decay-accelerating factor (DAF) contribute to actin remodelling of the cell cytoskeleton and viral delivery to the tight junction with subsequent cell entry [87,88]. Rotaviruses also gain cellular entry through cellular receptors, specifically the junctional adhesion molecules-A (JAM-A), triggering an entry pathway associated with this superfamily similar to coxsackievirus [82].

### 4.3. Cell Cytoskeleton Alteration

Some microorganisms increase GI permeability through the modulation of the epithelial cell cytoskeleton either by destabilising toxins, or triggering myosin/actin contraction by MLCK, Rho GTPases pathway activation [89,90,91].

The pathology of salmonellosis is associated with virulence factors’ intra-epithelial translocation to the host cell cytoplasm via the type 3 secretion system (T3SS), primarily SopB, SopE, SopE2 and SipA, that also play a role in RhoGTPase pathway activation and the subsequent actin dynamics [59,60,83,84]. *E. coli* also uses T3SS to inject secreted proteins, EspB, EspF, EspH and Tir, and induce actin/myosin contraction by activating MLCK processes [66,85]. Furthermore, fungi in the *Aspergillus* and *Penicillium* genera have been cited to alter the epithelial cell cytoskeleton by disrupting F-actin filaments, leading to an impaired cellular function and TJ structure [86].

The gastro-intestinal barrier can be challenged by a multitude of infectious agents that have developed a variety of mechanisms to disrupt normal GI function and permeability, mainly through the exploitation of tight junction structures. As concisely but not exhaustively described, the infectious aetiology can present dynamic strategies for addressing gut leakiness.

## 5. Leaky Gut and Diseases

Most diseases are multifactorial, with genetic predispositions and environmental triggers conspiring and initiating these diseases. Environmental variations, such as diet, pollution and hygiene, can lead to a dysbiotic gut. As discussed above, changes in microbiota composition can have detrimental effects on the gut epithelial barrier, increasing its permeability and allowing the translocation of bacteria and their products from the gut lumen, leading to systemic effects. This strong connection between an altered intestinal microbiome and a leaky gut is proposed to be the initiating event underlying a wide spectrum of disorders from inflammatory and autoimmune diseases to metabolic and neurological diseases (Table 2). In this section, we will address the involvement of the leaky gut in the pathogenesis of these diseases, as well as therapy-induced gut complications.

### 5.1. Inflammatory Bowel Diseases (IBDs)

IBDs include ulcerative colitis (UC) and Crohn’s disease (CD), and both diseases have a similar complex and a not fully defined aetiology. However, there is strong evidence that epithelial barrier dysfunction contributes to the onset of these diseases. Disrupted expression, cellular localisation and the function of tight junction proteins are key contributors to disrupted barriers in IBDs. Whereas the expression of some tight junction proteins, such as occludin, is reduced in IBD patients [92], the expression of others is upregulated, like claudin 1 and claudin 2 [93]. This increased expression can be attributed to the effect of the inflammatory cytokine IL-6, which is found in abundance in IBD patients [124]. Conversely, the microRNA miR-195-5p, which is reduced in UC patients in comparison to healthy controls, can impede the expression of these two claudins [125]. Furthermore, miR-24 is another microRNA that has been revealed to be specifically elevated in the colon tissue and even the blood of UC patients. This upregulated expression of miR-24 was shown to compromise epithelial barrier function in vitro by reducing the expression of cingulin, a tight junction-associated protein that is also downregulated in UC patients [94]. In addition to forming a protective physical barrier, intestinal epithelial cells play a role by influencing immune function. Aryl hydrocarbon receptor (AhR) signalling in the gut epithelia was shown to be indispensable for the induction and colonic accumulation of regulatory T cells (Tregs), following treatment with *Indigo naturalis*, which ameliorates the disease in UC patients and in a DSS-induced colitis mouse model [126].

Irregularities in the microbiome have been linked to IBDs. Higher numbers of bacteria were identified in the intestinal mucosal layer of IBD patients when compared to healthy individuals [95]. These changes in the microbiome can also have an impact on tight junction proteins. *Fusobacterium nucleatum*, found in abundance in IBD patients, has a damaging effect on the intestinal epithelial barrier by regulating the expression and distribution of the tight junction proteins ZO-1 and occludin. This damaging effect was demonstrated by the promoted colitis in DSS-treated mice receiving *F. nucleatum* [96]. A multiomics study of two IBD patient cohorts revealed that a subset of UC patients displayed an abundance of proteases originating from the bacterial species *Bacteroides vulgatus*, which disrupted the epithelial barrier by altering the tight junction proteins ZO-1 and occludin in vitro. Protease inhibitors prevented disease development in IL-10-deficient mice colonised by *B. vulgatus* [97]. Moreover, host-derived matrix metalloproteinases (MMPs) have been shown to be expressed at higher levels in IBD patients in comparison to healthy controls [127]. It has been demonstrated that inflammatory factors drive the expression of MMP-7, which is present at high levels in UC patients and rodent models of colitis [98]. Through targeting the degradation of the tight junction protein claudin-7, MMP-7 increases gut epithelial permeability and consequently aggravates the ongoing inflammation, which was ameliorated in MMP-7-deficient mice or by using MMP-7-blocking antibodies [98].

Microbiome-derived metabolites are also known to affect epithelial barrier fitness. In a longitudinal study in both UC and CD patients showed a reduction in the tryptophan metabolite indole-3-propionic acid (IPA) in UC patients in comparison to non-IBD individuals [99]. IPA is well known for its role in maintaining a healthy barrier [44]. A bacterial consortium used for the treatment of UC (mouse model) had an immunological effect and showed increased concentrations of IPA and butyrate [128]. Other tryptophan metabolites, like xanthurenic (XANA) and kynurenic (KYNA) acids, are negatively correlated with inflammation severity both in human IBD patients and mouse DSS models [129]. Supplementing mice with these two metabolites enhanced the viability and proliferation of epithelial cells, as well as modulating T-cell responses, leading to ameliorated disease severity in the DSS model [129].

Host-generated bile acids are also documented to influence the gut epithelial barrier. Cholic acid, one of the major bile acids produced by the liver, has been found to be more abundant in IBD patients and colitic mice due to the induction of cytochrome P450 8B1 (CYP8B1), which synthesises this bile acid. By inhibiting peroxisome proliferator-activated receptor alpha (PPARα), cholic acid hindered fatty acid oxidation and blocked intestinal stem cell renewal. The importance of this pathway is demonstrated in *Cyb8b1*-deficient mice, which are resistant to induced colitis [100]. Furthermore, chenodeoxycholic acid (CDCA) is another primary bile acid with detrimental effects on the epithelial barrier.

A dysfunctional actin cytoskeleton can also be the underlying reason for a defective barrier. Indeed, epithelial-specific abrogation of non-muscle myosin II, a cytoskeletal protein that regulates actin distribution and dynamics, causes alterations in tight junction proteins’ localisation, leading to compromised barrier function and mucosal inflammation [130]. Actin crosslinking factor 7 (ACF7) is another key player in cytoskeletal stability. Loss of ACF7 in Caco-2 cells has been demonstrated to destabilise tight junction proteins. In mice, intestinal-specific ACF7 knockout renders these mice more susceptible to DSS-induced colitis. Interestingly, the expression of the *ACF7* gene has been shown to be reduced in UC patients, when compared to healthy controls, implying its potential role in the disease pathology [101]. Furthermore, a deficiency of geranylgeranyltransferase type 1 (GGTase1), a phenyltransferase, in epithelial cells in mice causes cytoskeleton rearrangements and consequently arrested cell shedding, eventually leading to increased gut permeability and ensuing immune activation [102]. In another study, a subset of IBD patients exhibited high expression levels of tripartite motif-containing protein 40 (TRIM40), which is epigenetically silenced in healthy controls. Downstream signalling of TRIM40 is detrimental for cortical actin formation and stabilisation, leading to compromised barrier function and subsequently persistent inflammation. TRIM40 deficiency protects mice from DSS-induced colitis, further demonstrating the key role of this protein [131]. Another example of the importance of epigenetic regulation of epithelial functions is the association between IBDs, particularly CD, and genetic variants in the DNA methyltransferase 3 A (DNMT3A) [103], which plays an important role in de novo DNA methylation [132]. DNMT3A expression is reduced in gut epithelial cells of CD patients, in comparison to healthy controls, and in mouse intestinal organoids following exposure to tumour necrosis factor (TNF). Mice with DNMT3A-deficient epithelial cells display structural alterations in the gut epithelia, as well as increased colonic permeability, rendering these mice more prone to experimentally induced colitis [133]. Finally, microbiome-derived butyrate was demonstrated to epigenetically regulate the expression of synaptopodin, an actin-binding protein that localises to epithelial tight junctions, playing a critical role in barrier function and cell motility [134].

The maintenance of healthy intestinal epithelial barrier functions relies heavily on autophagy, which regulates different physiological aspects [135]. Several genes within the autophagy pathway, such as autophagy-related 16-like 1 (*ATG16L1*) [104,105,106], immunity-related GTPase M (*IRGM*) [107,108], unc51-like autophagy-activating kinase 1 (*ULK1*) [108,109], and leucine-rich repeat kinase 2 (*LRRK2*) [110] are associated with susceptibility to CD. Furthermore, an association between IBDs and the autophagy factor and transcriptional regulator brahma-related gene 1 (BRG1) has been demonstrated [111]. Indeed, BRG1 mRNA and protein colonic expression was shown to be downregulated in UC and CD patients in comparison to healthy controls. Through regulating the transcription of autophagy factors, BRG1 regulates the levels of reactive-oxygen species (ROS), which at high levels lead to epithelial cell apoptosis and defective barrier functions. Mice with intestinal epithelial-specific deletion of BRG1 develop spontaneous colitis and demonstrate disrupted barrier functions and increased permeability, further emphasising the role of BRG1 [111]. Intestinal epithelial autophagy can be influenced by microbiome-derived mediators. The colonic protective effects of butyrate against DSS-induced injury have been attributed to its ability to regulate epithelial cell autophagy via hypoxia-inducible factor-1α (HIF-1α) [136]. Furthermore, UC patients exhibit elevated levels of autotaxin, a secreted glycoprotein that amplifies barrier disruption by inhibiting mammalian target of rapamycin (mTOR)-dependent autophagy [137]. The inhibition of autotaxin and the administration of rapamycin ameliorated colonic inflammation and reversed epithelial barrier damage in DSS-treated mice [137].

In conclusion, all factors impacting epithelial barrier health, whether intrinsic, like autophagy, or extrinsic, like the microbiome, can contribute to IBD pathology.

### 5.2. Rheumatic Diseases

The concept of the gut–joint axis has recently emerged following the strong evidence of gut involvement in the aetiology of rheumatic diseases. Indeed, gut microbiome dysbiosis has been described in a wide variety of rheumatic diseases, such as rheumatoid arthritis (RA), ankylosing spondylitis (AS), psoriatic arthritis (PsA) and systemic lupus erythematosus (SLE) [138]. The importance of the gut microbiome in RA initiation has been demonstrated in different RA mouse models, where mice raised under germ-free conditions only develop mild autoimmune arthritis, in comparison to specific-pathogen free (SPF) mice [139,140]. Interestingly, colonising those germ-free mice with specific bacterial species, such as segmented filamentous bacteria [140], *Lactobacillus bifidus* [139] and *Subdoligranulum didolesgii* [141] was sufficient for disease induction.

In addition to modifying immune functions, gut dysbiosis is hypothesised to contribute to RA pathology by disrupting the gut barrier function, which would facilitate the translocation of bacteria or their components/products into the lamina propria, spreading systemically and eventually leading to inflammatory responses [142]. Impaired barrier function has been reported in individuals with pre-clinical RA, early onset RA and fully established RA [112,143]. This is demonstrated by the reduced expression of the tight junction proteins occludin and claudin-1 in the intestinal epithelium [112] and increased circulating concentrations of LPS and LPS-binding protein in RA patients [143] when compared to healthy controls. This was accompanied by increased gut intestinal permeability in these patients [112]. In connection to facilitated bacterial trafficking through the gut barrier, bacterial DNA and bacterial wall components were traced in the synovial fluid of RA patients [144,145,146]. 16S rRNA gene sequencing of RA patients’ synovial fluid samples revealed that this joint invasion occurs mainly in advanced stage 4 RA (RAS4) patients, with the highly RA-associated species *Prevotella copri* [146,147,148] being found in most of these samples. Scanning electron microscopy analysis of the synovial fluid samples of RAS4 patients revealed objects with a rod-like or spheric shapes, and some bacterial species were successfully cultured in a fraction of these samples, clearly indicating the active bacterial invasion of the joints [146].

The observed alterations in intestinal barrier functions, associated with RA, can be attributed to changes in gut bacteria. For instance, the bacterial genus *Collinsella*, found in abundance in RA patients, can compromise the gut barrier [113,149,150]. *Collinsella aerofaciens* that has been reported to reduce the expression of the tight junction proteins ZO-1 and occludin in the Caco-2 cell line and increase disease severity in collagen-induced arthritis-susceptible HLA-DQ8 mice [113]. Similarly, the increased abundance of *Enterobacteriaceae* in RA patients [114] could contribute to amplified inflammation by increasing gut permeability, as previously reported in diabetic patients and healthy individuals with reduced barrier function [123]. Conversely, a lower abundance of *Bifidobacterium adolescentis*, *Bifidobacterium longum* and *Faecalibacterium prausnitzii*, all known for their gut barrier protective properties [151,152,153,154,155], has been reported in RA [114]. Interestingly, restoring barrier function via the administration of *Bifidobacterium adolescentis* [156] or butyrate [112] was shown to mitigate arthritis in rodent models. RA patients responding to biological disease-modifying antirheumatic drugs (bDMARDs) also displayed improved gut barrier function, demonstrated by a reduction in circulating permeability markers [157]. Collectively, all of these data indicate the key role of intestinal barrier function in the initiating events and pathology of RA.

Patients with AS or PsA have been reported to exhibit dysbiotic intestinal microbiota, as well as subclinical gut inflammation [115,116,117], implying a role of the microbiome in disease pathogenesis. Furthermore, healthy individuals with the HLA-B27 risk allele, associated with AS, display altered microbial composition [158]. Raising *HLA-B27* transgenic rats under germ-free conditions was reported to protect against the spontaneous development of gastrointestinal and joint inflammation, further supporting the potential role of the microbiome in AS [159,160]. Furthermore, AS patients were reported to have a reduced gut epithelial expression of the tight junction proteins claudin 1, claudin 4, occludin and ZO-1, accompanied by increased gut permeability and surging systemic concentrations of permeability markers (LPS and LBP) [118]. These findings suggest that gut dysbiosis leads to a deteriorating gut barrier function, initiating inflammatory events in AS. Interestingly, restored gut barrier integrity and tight junction protein expression were associated with reduced disease activity, following the application of the tryptophan metabolite indole-3-acetic acid in an AS mouse model [161].

In SLE, the enhanced concentrations of patients’ faecal calprotectin, a widely recognised marker of increased gut permeability [162], indicate the role of intestinal barrier impairment in SLE development [163,164]. This role is further corroborated in mice, where inducing gut leakiness in two murine models of lupus, FcgRIIb^−/−^ and pristane-induced, intensified disease progression [119]. The increased gut permeability in these models was found to promote the translocation of gut microbial components, as demonstrated by higher endotoxin and β-glucan serum concentrations and a higher bacterial burden in mesenteric lymph nodes (MLNs), which induced host cell apoptosis and eventually triggered the production of anti-dsDNA autoantibodies and immune complex formation, leading to disease aggravation [119]. Another study demonstrated that microbiome dysbiosis in a MRL/Ipr lupus mouse model was associated with increased colonic oxidative stress and intestinal permeability, leading to inflammatory responses [165]. Interestingly, significant disease amelioration in mouse models, following treatment with antibiotics, probiotics or antioxidants, was associated with an improved intestinal barrier, providing further evidence of the role of barrier function in SLE [165,166,167].

Altogether, there is a strong link between different rheumatic diseases and the integrity of the gut epithelial barrier and the interaction of the latter with the gut microbiome. Further understanding of this three-way relationship may prompt novel therapies of these diseases.

### 5.3. Metabolic Diseases

High-fat diets (HFD) are major drivers of prevalent metabolic diseases, like type 2 diabetes and non-alcoholic fatty liver disease (NAFLD), which are characterised by underlying low-grade inflammation that can be linked to a leaky gut. Indeed, dietary fats have been demonstrated to have a direct impact on epithelial barrier integrity. For instance, exposing obese rat strains to a HFD for 16 weeks led to a reduction in the expression of intestinal tight junction proteins, such as claudin-1, claudin-3 and junction adhesion molecule (JAM)-1, which correlated with increased gut permeability [120]. Similarly, mice exposed to a prolonged HFD not only displayed classic metabolic manifestations, such as glucose intolerance and body weight gain, but also elevated intestinal permeability, which correlated with a reduced expression of the tight junction protein ZO-1. Notably, these effects were reversed by the administration of wide-spectrum antibiotics in combination with HFD, indicating the involvement of gut microbiota in mediating the impact of HFDs [168].

Similar observations of compromised barrier function and increased bacterial translocation were confirmed in obese leptin receptor-deficient mice [121]. However, obesity per se was not identified as a cause of barrier dysfunction, which was attributed to hyperglycaemia; a common feature between obesity and HFD models [121]. This link was further corroborated in a streptozotocin (STZ)-induced type 1 diabetes model, which displayed deteriorating barrier function that could be mitigated by the application of insulin [121]. An in vitro analysis of the Caco-2 epithelial cell line revealed that exposure to increasing glucose concentrations altered epithelial cell functions and barrier integrity. This is facilitated by the bidirectional glucose transporter GLUT2, as the epithelial-specific deletion of GLUT2 protected against the STZ-induced reprogramming of epithelial cells and the loss of expression of tight junction proteins [121]. In line with these findings, high-glucose (HGD) and high-fructose (HFrD) diets were revealed to cause glucose intolerance and increase barrier permeability, attributed to a reduced expression of occludin and ZO-1, as well as an increased expression of inflammatory mediators [122]. In line with these findings, a recent study highlighted a correlation between a compromised gut barrier, determined by the reduced expression of mucins and antimicrobial peptides, and a reduced abundance of short chain fatty acids-producing bacteria, like *Bifidobacterium dentium*, *Clostridium butyricum* and *Roseburia intestinalis* [169]. Altogether, systemic elevation of glucose concentrations, achieved by means of different methods, is a major cause of gut epithelial barrier disruption in metabolic diseases.

The diet-induced impairment of barrier function can also be caused by changes in the microbiome’s makeup. In addition to barrier disruption, a HFD was found to introduce dramatic changes in the mouse caecal microbiome composition, in comparison to a normal diet, with a strong reduction in the abundance of *Lactobacillus* species, *Bifidobacterium* species and *Bacteriodes-Prevotella* species [168]. Furthermore, a HFD was revealed to favour a higher abundance of hydrogen sulphide-producing bacteria, compared to a normal diet, which was associated with poorer colonic transepithelial resistance [170]. Similarly, mice subjected to a HGD and HFrD displayed a lower abundance of *Bacteriodetes* and an increase in *Proteobacteria* in comparison to mice fed with a normal diet, which was associated with a disrupted barrier [122]. Finally, the Gram-negative order *Enterobacteriales* was reported to be enriched in type 2 diabetes patients and healthy controls with high colonic permeability, indicating its contribution to disease development by impairing barrier functions [123].

Dietary fat may indirectly modulate gut barrier function by promoting bile acid production. This was demonstrated in mice fed with HFD, which prompted hyperpermeability in jejunum and colon. This correlated with an increase in the faecal concentrations of almost all bile acids [171]. This is supported by previous studies investigating the effect of bile acids on epithelial cells in vitro. Cholic acid has been shown to reduce the electric transepithelial resistance (TEER) of Caco-2 cell monolayers, implying compromised barrier function. This effect was mediated by increasing the production of ROS in epithelial cells [172]. This was corroborated by another study that showed similar effects being exerted by cholic acid, deoxycholic acid (DCA) and CDCA, which led to increased permeability of Caco-2 cell monolayers, led by epidermal growth factor receptor (EGFR) autophosphorylation and occludin redistribution in tight junctions [173].

## 6. Therapy-Induced Epithelial Barrier Dysfunction

In this section, we will cover gut epithelial barrier disruption resulting from certain therapeutics and the mechanisms of action and consequences of those disruptions. We will not discuss immunotherapy-related colitis, which is the outcome of T-cell hyperactivity, leading to inflammatory colitis, rather than the direct disruption of the gut epithelium.

### 6.1. Radiation Enteritis

Radiation enteritis is a bowel injury resulting from radiotherapy of malignancies in the abdominal or pelvic regions. It affects almost 90% of patients undergoing radiotherapy, with up to 10% suffering from severe forms of enteritis [174]. The high turnover rates of the small intestinal and colonic epithelia, estimated to be the highest amongst solid tissues [175], render them prone to radiation injury. This sensitivity is manifested in the well-documented radiation-induced increase in gut epithelial permeability and abrogation of the expression of tight junction protein expression [176,177]. The interplay between the intestinal epithelial barrier and the microbiome is believed to be the major contributor to radiation enteritis. Indeed, germ-free mice are resistant to radiation enteritis [178]. Consistently, antibiotic treatment prior to radiation was found to improve gut injury through microbiome remodelling and consequently inflammation inhibition [179]. Moreover, gut microbiome dysbiosis has been associated with radiation enteritis in patients exposed to pelvic radiation. Patients suffering from radiation enteritis displayed an increased abundance of *Proteobacteria* and *Gammaproteobacteria* and a lower abundance of *Bacteroides*. Upon co-culture with colon epithelial cells, the dysbiotic microbiome from radiation enteritis patients led to disrupted barrier function and the induction of inflammatory cytokines [180]. Dysbiosis correction through faecal microbiota transplantation (FMT) has been revealed to protect against radiation-induced gut injury in a radiation mouse model. This was demonstrated by enhanced barrier function and an increase in mucin production [181]. Interestingly, the efficacy of FMT in relieving radiation-induced enteritis was demonstrated in a pilot study with cancer patients. Three out of the five treated patients responded by displaying improvement in symptoms following FMT application, which was completely safe [182]. The protective effect of the microbiome can be attributed to certain bacterial metabolites that enhance epithelial barrier function. Supplementing mice undergoing abdominal irradiation with IPA, a microbiome-derived tryptophan metabolite, was shown to mitigate radiation-induced gut injury and restore the gut barrier function [183]. In a different study, IPA was shown to improve barrier function by increasing the expression of tight junction proteins and mucins when applied to the epithelial cell lines Caco-2 and HT29 [44]. In conclusion, structuring the microbiome to be supportive of the gut epithelial barrier by means of the administration of antibiotics, FMT or any form of live biotherapeutic products (LBPs) is the way forward for the treatment of radiation enteritis.

### 6.2. Chemotherapy-Induced Gut Toxicity

One of the major adverse effects of cancer chemotherapy is gastrointestinal tract toxicity, causing nausea, vomiting and diarrhoea. These effects are attributed to chemotherapy-induced disruption of the structure and function of the epithelial barrier. Chemotherapeutic agents can stimulate enteroendocrine cells to produce mediators, such as prostaglandins and 5-hydroxy tryptamine, which trigger an emetic response by activating the vagal nerve [184]. Furthermore, chemotherapy-induced histological damage, including crypt ablation, epithelial atrophy and villi blunting, is highly regarded as the driver of the accompanying diarrhoea [185,186].

In addition to clinical manifestations, chemotherapy can lead to bacterial translocation from the gut into the blood stream due to the disrupted gut epithelial barrier. The ensuing infections are central to chemotherapy-associated morbidity and mortality, particularly in children’s haematological malignancies [187]. It has become clear that the gut microbiome plays a significant role in this serious complication. Indeed, the abundance of *Enterococcaceae* or *Streptococcaceae* at any stage of chemotherapy can predict infections in children treated for acute lymphoblastic leukaemia (ALL) [188]. This was further supported by another study, demonstrating clear differences in microbiome composition between ALL patients contracting pneumonia, following chemotherapy, and unaffected ALL patients [189]. Altogether, chemotherapy-induced barrier disruption not only resulted in gastrointestinal symptoms, but also, in combination with a certain microbiome makeup, can lead to life-threatening infections.

### 6.3. Graft-versus-Host Disease

Graft-versus-host disease (GVHD) is a common complication of allogenic hematopoietic stem cell transplantation, whereby donor T cells target the recipient’s antigens, leading to tissue damage. MHC-II antigen presentation by IEC is the initiating event of gastrointestinal GVHD. Indeed, gut epithelial damage, resulting from pretransplant conditioning such as radiation, has been shown to be important for the initiation of GVHD [190]. Furthermore, loss of gut barrier function is postulated to support the propagation phase of GVHD, as a poorer intestinal barrier was demonstrated to correlate with disease severity [191,192]. This was further corroborated by observing that long myosin light-chain kinase (MLCK210), a key regulator of tight junction permeability, is elevated in human GVHD biopsies [193]. Transplant-receiving mice that lack MLCK210 displayed less disease propagation [193]. This clearly demonstrates that increased gut permeability, dependent on MLCK210, is required for GVHD propagation. Another important element within the epithelial barrier is goblet cells, responsible for mucus layer formation. Loss of goblet cells is characteristic of severe GVHD and results in the disruption of the inner mucus layer and increased bacterial translocation [194]. This is supported by observing the beneficial effects of the administration of IL-25, a goblet cell growth factor, prior to transplantation in ameliorating GVHD [194].

The gut microbiome has been revealed to contribute to the development of GVHD, as germ-free mice are partially protected from disease development [195], and antibiotic treatment showed clinical benefits in humans [196]. This implies that microbiome–barrier interactions could play a role in GVHD. Indeed, MHC-II expression by intestinal epithelial cells, required for antigen presentation and the activation of donor CD4+ T cells, is dependent on gut microbiota, as IECs from germ-free mice lacked MHC-II [197]. Moreover, donor T cells not only target intestinal stem cells but also innate lymphoid cells (iLC), which mediate intestinal barrier recovery following pre-transplant conditioning through the production of IL-22 [198]. Collectively, the interplay between the gut epithelial barrier, the microbiome and the immune system is important for the initiation and progression of GVHD.

## 7. Concluding Remarks

The interaction of the intestinal microbiome with the gut epithelium has a significant impact on the integrity of the barrier, as outlined above. The disturbance of this axis can lead to a loss of barrier integrity during enteric infections, with *V. cholerae* and enteropathogenic *E. coli* being extreme examples. Non-pathogen-induced changes in the microbiome can also lead to gut leakiness and the unregulated escape of gut luminal contents from food antigens into bacterial products. This has been associated with local inflammation and pathology in intestinal diseases, such as IBDs and radiation enteritis, but also distal and/or systemic effects in other diseases including several autoimmune and metabolic diseases.

The human intestinal microbiome typically consists of trillions of bacteria belonging to hundreds of different species that vary among individuals, and each strain/species is likely to interact with the host via multiple mechanisms. Despite this complexity, the research discussed in this review demonstrates the considerable progress made in elucidating the mechanisms by which the microbiome affects the epithelial barrier. Undoubtedly, there are more mechanisms to be unravelled in health and disease. The most significant challenge to the field will be understanding how these mechanisms interact and working toward an integrated understanding of how the entire ecosystem leads to an overall positive or negative impact.

Given the contribution of the gut microbiome–epithelium axis to the pathology of several conditions, this axis should be a target for disease prevention and therapeutic intervention. Diet, probiotics and FMT can impact the microbiome to a greater or lesser degree and have been used in many of the above indications. Diet can have significant benefits in metabolic disease, such as reversing type 2 diabetes, but obviously the impact here is multifaceted. Beyond this, specific diets have had limited consistent therapeutic benefits in many of the other indications [199]. Personalising these diets based on an individual’s microbiome, as used to improve glucose control [200,201], may be the answer to achieve therapeutic benefit. Likewise, probiotics have modest effects at best in broad patient populations, despite preclinical data suggesting a positive benefit on barrier integrity [202]. FMT can impact the microbiome and has been shown to lead to a positive outcome in dysbiotic diseases, including IBDs [203]. Normalisation of a dysbiotic microbiome by FMT will have multiple effects on the patient, with restoring gut barrier integrity likely to be a key mechanism [204]. However, FMT can be challenging due to the availability of healthy donor stools, patient acceptability and the possibility of transferring adventitious agents including multidrug-resistant organisms, plus FMT is inconsistent, leading to the “super-donor” concept [205].

Beyond these non-specific approaches to modulating the microbiome, there is a paucity of medicines, including those in development, targeting the leaky gut. This review outlines the increasing appreciation of the full complexity of microbiome–epithelium interactions, which should allow for new medicines to be developed that target the axis more specifically. New microbiome modalities targeted at key bacterial species, such as consortia of beneficial commensal bacteria or bacteriophages to deplete negative species, are being developed for other mechanisms and could be developed for the leaky gut. In addition, a detailed understanding of the metabolites involved could enable new small molecule modulators of the axis.

The data discussed above clearly demonstrate that the interaction between the intestinal microbiome and the epithelium is incredibly important for the maintenance of gut barrier integrity and contributes to the leakiness that feeds into the pathology of several diseases. Current and future advances might inspire a new wave of therapeutics that could have benefits that cannot be realised by current medicines.

## Figures and Tables

**Table 1:** Examples of pathogen-induced modulation of gut permeability. CAR: coxsackievirus and adenovirus receptor; cGMP: cyclic guanosine monophosphate; CTA: cholera toxin subunit A; ERK1/2: extracellular signal-regulated kinase ½; Ha/P: hemagglutinin protease; HtrA: high temperature requirement protein A; JAM-A: junctional adhesion molecules-A; MLCK: myosin light-chain kinase; PKA: protein kinase A; Rho: RAS homolog; TJ: tight junction; ZOT: zonula occludens toxin.

Gut Permeability Alteration	Pathogen	Mechanism
Tight junction protein degradation and reorganisation	*Campylobater jejuni* [68,69]	Cleavage of occludin and E-cadherine proteins by HtrA
	*Clostridium perfringens* [68,69,70,71,72,73,74,75]	TJ destruction by direct enterotoxin binding with claudin 4
	*Entamoeba histolytica* [68,69,70,71,72,73,74,75]	TJ destruction by direct enterotoxin binding with claudin 1 and 2
	*Vibrio cholerae* [59,76,77,78]	CTA modulation of chloride ion channels
		ZOT modulation of TJ proteins in small intestine
		Degradation of TJ proteins and TJ morphology rearrangement by Ha/P proteinase
Alteration of host cell signalling pathways associated with TJ complex	Enteropathogenic *Escherichia coli* [79]	Occludin re-arrangement by cGMP and PKA formation triggered by enteropathogenic *E. coli* enterotoxin A
	*Shigella flexneri* [78,80]	Alteration of claudin 2 and 4, occluding and ZO-1 proteins by modulation of ERK1/2
	*Campylobacter concinus* [79,80,81]	TJ pathway regulation similar to *V. cholerae* ZOT
	*E. coli* [79,80,81]	
	*Coxsackievirus, Adenovirus* [81,82]	TJ disruption by occluding internalisation due to CAR
	*Rotavirus* [82]	TJ disruption and cellular entry due to JAM-A
Cell cytoskeleton alteration	*Samonella* sp. [59,60,83,84]	Actin alteration via SopB, SopE, SopE2, SipA and Rho GTPases pathway
	*E. coli* [66,85]	Actin/myosin contraction via EspF, EspH, Tir and MLCK processes
	*Aspergillus and Penicillium* [86]	F-actin filament disruption

**Table 2:** Summary of gut epithelial mechanisms, contributing to the pathology of inflammatory bowel diseases (IBDs), rheumatic diseases and metabolic diseases. ACF-7: actin crosslinking factor 7; AS: ankylosing spondylitis; BRG1: brahma-related gene 1; CD: Chron’s disease; DNMT3A: DNA methyltransferase 3 A; DSS: dextran sodium sulfate; GGTase1: geranylgeranyltransferase type 1; HFD: high-fat diet; IBD: inflammatory bowel disease; IL-10: interleukin 10; IPA: indole-3-propionic acid; JAM-1: junction adhesion molecule-1; MMP-7: matrix metalloproteinases-7; PsA: psoriatic arthritis; RA: rheumatoid arthritis; ROS: reactive oxygen species; SLE: systemic lupus erythematosus; UC: ulcerative colitis; ZO-1: zonula occludens-1.

	Changes in Gut Epithelial Barrier	Patients/Experimental Models
Inflammatory bowel diseases	Occludin downregulation [92]	IBD patients
	Claudin 1 and claudin 4 upregulation [93]	IBD patients
	Cingulin downregulation [94]	UC patients
	Higher bacterial counts in mucosal layer [95]	IBD patients
	*F. nucleatum* regulates the expression and distribution of ZO-1 and occludin [96]	IBD patients/DSS model
	*B. vulgatus* alters ZO-1 and occludin [97]	IBD patients/IL-10 deficient mice
	Claudin 7 degradation by MMP-7 [98]	Colitis mouse model
	Reduction in the level of IPA, which improves barrier function [44,99]	IBD patients
	Downregulation of cholic acid, which is important for intestinal stem cell renewal [100]	IBD patients/colitic mice
	Reduced expression of ACF7, important for cytoskeletal stability [101]	UC patients/DSS mouse model
	GGTase1 deficiency, leading to increased permeability [102]	Mouse model
	Reduced expression of DNMT3A, important for epithelial structural regulation [103]	CD patients/mouse
	Autophagy, important for regulation of barrier functions, are associated with CD susceptibility [104,105,106,107,108,109,110]	CD patients
	Downregulated colonic expression of BRG1, important for regulating ROS levels and protecting the epithelial barrier [111]	IBD patients/mouse
Rheumatic diseases	Reduced expression of occludin and claudin-1 [112]	RA patients
	*Collinsella aerofaciens* reduces the expression of ZO-1 and occludin and increase disease severity [113]	In vitro/mouse RA model
	Increased abundance of *Enterobacteriaceae* increase gut permeability [114]	RA patients
	Reduced abundance of barrier protective *Bifidobacterium adolescentis*, *Bifidobacterium longum* and *Faecalibacterium prausnitzii* [114]	RA patients
	Subclinical gut inflammation and dysbiosis [115,116,117]	AS and PsA patients
	Reduced epithelial expression of claudin 1, claudin 4, occludin and ZO-1 [118]	AS patients
	Increased gut permeability intensifies disease severity [119]	SLE mouse model
Metabolic diseases	Reduced expression of claudin-1, claudin-3 and JAM-1, and increased gut permeability [120]	HFD-fed mouse model
	Hyperglycaemia alters barrier integrity [121]	Mouse model/in vitro
	High glucose and high fructose diets increased gut permeability and reduce the expression of occludin and ZO-1 [122]	Mouse model
	Increased abundance of *Enterobacteriales*, correlated with increased colonic permeability [123]	Type II diabetes patients
